# Five-year comparison of clinical and echocardiographic outcomes of pure aortic stenosis with pure aortic regurgitation or mixed aortic valve disease in the COMMENCE trial

**DOI:** 10.1016/j.xjon.2024.08.020

**Published:** 2024-09-10

**Authors:** Vinod H. Thourani, John D. Puskas, Bartley Griffith, Lars G. Svensson, Philippe Pibarot, Michael A. Borger, David Heimansohn, Thomas Beaver, Eugene H. Blackstone, Anna Liza M. Antonio, Joseph E. Bavaria, COMMENCE Trial Investigators

**Affiliations:** a Department of Cardiovascular Surgery, Marcus Valve Center, Piedmont Heart Institute, Atlanta, Ga; b Division of Cardiothoracic Surgery, Emory University School of Medicine, Atlanta, Ga; c Department of Surgery, University of Maryland Medical Center, Baltimore, Md; d Department of Thoracic and Cardiovascular Surgery, Cleveland Clinic, Cleveland, Ohio; e Department of Cardiology, Québec Heart and Lung Institute, Laval University, Quebec, Canada; f University Department of Cardiac Surgery, Heart Center Leipzig, Leipzig, Germany; g Department of Cardiothoracic Surgery, St. Vincent Heart Center of Indiana, Indianapolis, Ind; h Division of Cardiovascular Surgery, University of Florida Health, Gainesville, Fla; i Edwards Lifesciences, Irvine, Calif; j Department of Cardiovascular Surgery, Jefferson Health, Philadelphia, Pa

**Keywords:** aortic stenosis, aortic regurgitation, mixed aortic valve disease, aortic valve replacement, clinical outcomes

## Abstract

**Objective:**

To compare outcomes of aortic valve replacement (AVR) in patients with pure aortic stenosis (Pure AS) and those with pure aortic regurgitation (Pure AR) or mixed AS and AR (MAVD) in the COMMENCE trial.

**Methods:**

Of 689 patients who underwent AVR in the COMMENCE trial, patients with moderate or severe AR with or without AS (Pure AR + MAVD; n = 135) or Pure AS (n = 323) were included. Inverse probability of treatment weighting Kaplan-Meier survival curves were used for time-to-event endpoints, and longitudinal changes in hemodynamics were evaluated using mixed-effects models. Echocardiographic outcomes were assessed by an echo core laboratory and clinical outcomes adjudicated by a clinical events committee. The mean duration of follow-up was 5.3 ± 2.2 years.

**Results:**

At 5 years, adjusted safety endpoints were not statistically different between groups; no structural valve deterioration (SVD) event occurred in either group. After adjustment, the Pure AR + MAVD group had a greater change in body surface area–corrected left ventricular (LV) mass reduction (*P* = .03) compared to the Pure AS patients. Those patients with a baseline LV ejection fraction (LVEF) >55% continued to demonstrate preserved contractility compared to patients with an LVEF ≤55% at baseline (*P* < .0001). No significant difference in mean gradient (*P* = .07) or effective orifice area (*P* = .96) at 5 years was evident between the groups.

**Conclusions:**

Patients with Pure AR + MAVD demonstrated similar clinical safety and freedom from SVD at 5 years compared to those with Pure AS. There was a significant difference in LV reverse remodeling in the Pure AR + MAVD group compared to the Pure AS group at 5 years. These favorable outcomes in patients with AR may reinforce the need for treatment before irreversible changes occur.

## Graphical Abstract

**Figure:**
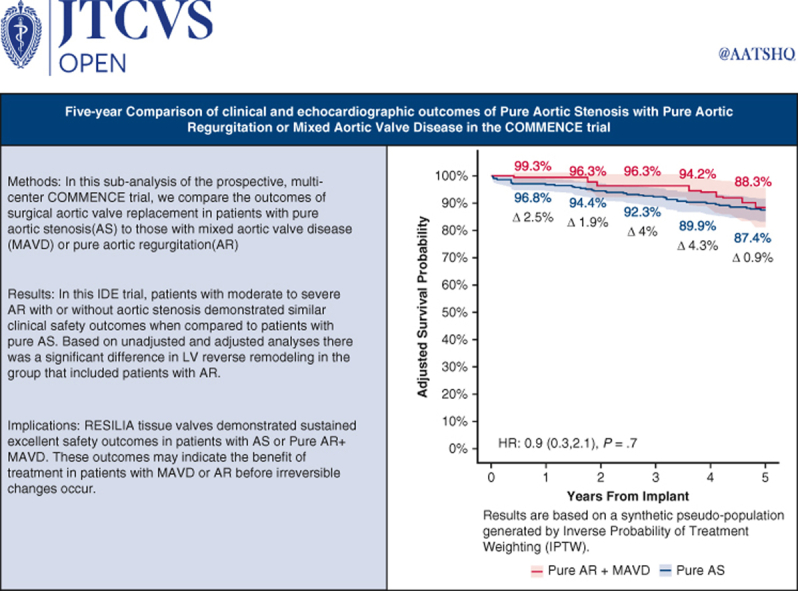

**Figure:**
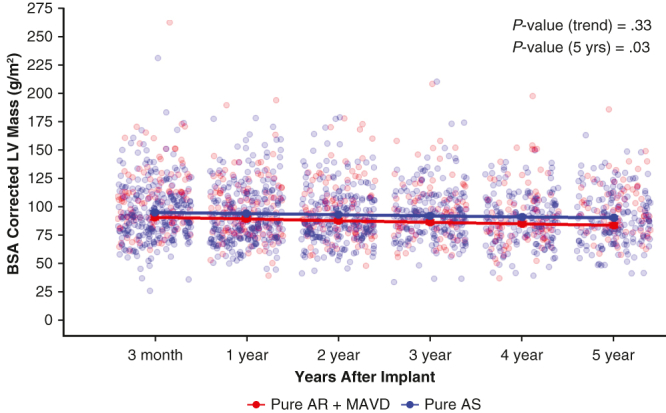
Body surface area (BSA)-corrected left ventricular (LV) mass for patients with pure aortic stenosis (Pure AS) compared to pure aortic regurgitation (Pure AR) or mixed aortic valve disease (MAVD) at 5 years (*P* = .03).

**Central Message**

At 5 years, patients with moderate to severe aortic regurgitation with or without aortic stenosis demonstrated significant left ventricular reverse remodeling after aortic valve replacement with RESILIA tissue.

**Perspective**

The COMMENCE aortic trial demonstrated similar clinical safety and hemodynamic outcomes in patients with moderate to severe aortic regurgitation compared to patients with pure aortic stenosis through 5 years.

See Discussion on page 174.

The timing of surgery and outcomes associated with pure aortic stenosis (AS) or aortic regurgitation (AR) have been studied, and treatment algorithms have been developed.^1^ While concentric left ventricular (LV) hypertrophy is common in patients with severe AS and eccentric remodeling is noted in patients with severe AR, the pathophysiology of mixed aortic valve disease (MAVD) remains less well studied. Surgical or transcatheter aortic valve replacement (AVR) is now a well-established technique for treating AS, while surgery alone is the most commonly used treatment for AR. However, the diagnosis and treatment triggers for surgery in those with AR or MAVD remain topics of debate. In cases where either AS or AR is singularly predominant, management should follow the guidelines for the predominant lesion^1^; however, when there is a more balanced disease process with complex pathophysiology and echocardiography-derived assessment, treatment pathways are less clear.

Recently published data suggest that the addition of AR in those with AS is associated with changes in echocardiographic pathophysiology leading to adverse outcomes.^2^ This may result in less improvement in LV function and functional capacity.^2^, ^3^, ^4^, ^5^ In this present study, we aimed to evaluate the 5-year clinical and echocardiographic outcomes in patients with Pure AS or Pure AR + MAVR who underwent surgical AVR in the prospective COMMENCE trial.

## Patients and Methods

### Study Design and Patients

The COMMENCE trial is a prospective, multicenter, single-arm Food and Drug Administration Investigational Device Exemption study designed to evaluate the safety and effectiveness of a valve with RESILIA tissue (ClinicalTrials.gov identifier NCT01757665). Patient inclusion and exclusion criteria have been reported previously.^6^, ^7^, ^8^ The present subanalysis included patients with mild, moderate, or severe AS and no regurgitation at baseline (Pure AS group) and patients with moderate or severe AR at baseline with or without AS (Pure AR + MAVD group). Patients with a diagnosis of replacement of “prosthetic valve dysfunction” were excluded.

The outcome for this subanalysis was all-cause mortality at 5 years. The secondary outcomes included echocardiographic outcomes. All safety endpoints included in the subanalysis were adjudicated by an independent Clinical Events Committee (CEC).^6^^,^^7^ These safety endpoints and hemodynamic performance were evaluated with Doppler echocardiography by an independent core laboratory (BioTelemetry Research). Evidence of LV reverse remodeling was measured by examining changes in LV dimensions over time. All patients underwent local informed consent for the COMMENCE trial. Institutional Review Board approval was provided at each study site (Appendix E1), and all study participants gave written consent for the publication of their study data.

### Data Management and Statistical Methods

The investigational sites were responsible for collecting and recording the clinical data. Edwards Lifesciences monitored, aggregated clinical data, and performed statistical analyses.

Variables are summarized as mean and standard deviation or median and interquartile range for continuous variables and as frequency and percentage for categorical variables. The Shapiro-Wilks test was used determine the normality of the data. The Wilcoxon rank-sum or signed-rank test was used for continuous variables. The χ^2^ or Fisher exact test was used for categorical variables. The Kaplan-Meier method and log-rank test were used for time-to-event endpoints. Longitudinal changes in hemodynamic measures postimplantation were evaluated using mixed-effects models. To assess patient–prosthesis mismatch (PPM) at 3 months (the earliest postoperative visit), PPM was defined as severe (indexed effective orifice area [EOAi] ≤0.65 at body mass index [BMI] <30 or ≤0.55 at BMI ≥30), moderate (EOAi >0.65 to ≤0.85 at BMI <30 or >0.55 to ≤0.70 at BMI ≥30), or none/mild (EOAi >0.85 at BMI <30 or >0.70 at BMI ≥30) (VARC3 definition of PPM). We then identified patients with severe PPM, elevated mean gradient (>20), and valve dysfunction (Doppler velocity index [DVI] <0.25).^9^

To appropriately consider potential differences in baseline characteristics between groups, a balancing score was used in the adjusted analysis (as either inverse probability of treatment weighting [IPTW]-adjusted or main effect).^10^ The balancing score for each patient was defined as the probability of having Pure AR or MAVD as opposed to Pure AS. All variables in the balancing score were physician-reviewed for comprehensiveness and clinical relevance. Covariate balance was considered achieved at an absolute standardized mean difference of <0.2. Missing baseline information was assumed to be missing at random, and multiple imputation was performed to generate 25 datasets.^11^ The results from each imputed dataset were summarized according to Rubin's rules for parameter pooling.^12^, ^13^, ^14^, ^15^, ^16^

### Subgroup Analysis

Subgroup analysis of the Pure AR + MAVD group was performed to examine whether survival rates differed among those with none/mild, moderate, or severe AS and among between those with differing baseline LV ejection fraction (LVEF) values. A significance level of 0.05 was used, and no adjustment for multiplicity was made. R version 4.3.2 (R Core Team) was used for all statistical analyses.^17^

## Results

### Patients and Baseline Characteristics

**Figure 1:**
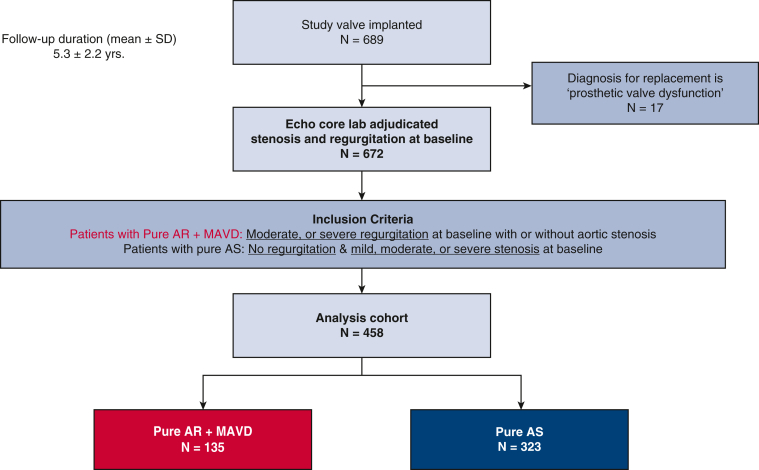
CONSORT diagram. *AR*, Aortic regurgitation; *MAVD*, mixed aortic valve disease; *AS*, aortic stenosis.

**Figure E1:**
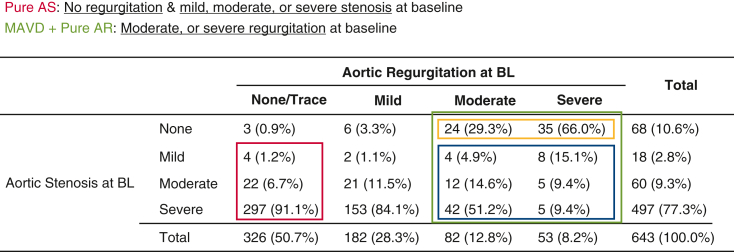
Derivation of the analysis cohorts. *AS*, Aortic stenosis; *MAVD*, mixed aortic valve disease; *AR*, aortic regurgitation; *BL*, baseline.

**Figure E2:**
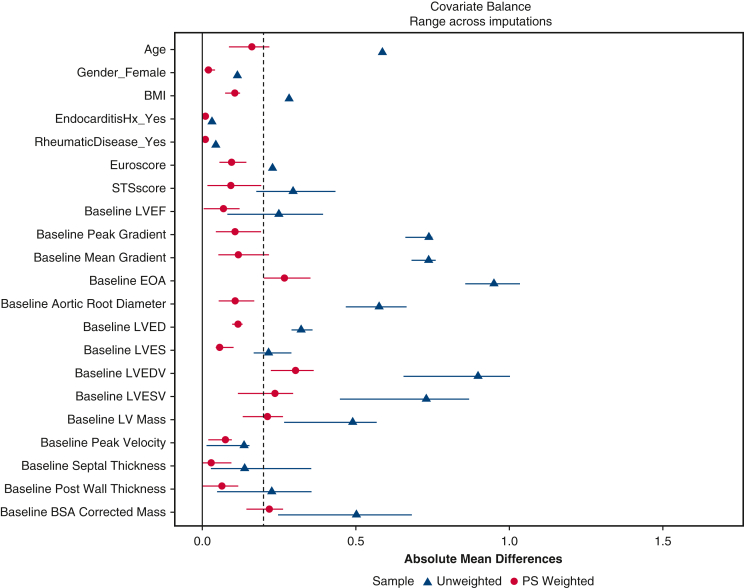
Love plot displaying covariate balance before and after inverse probability of treatment weighting and multiple imputation. *BMI*, Body mass index; *LVEF*, left ventricular ejection fraction; *EOA*, effective orifice area; *LVED*, left ventricular end diastolic dimension; *LVES*, left ventricular end systolic dimension; *LVEDV*, left ventricular end diastolic volume; *LVESV*, left ventricular end systolic volume; *BSA*, body surface area.

Of the 689 patients implanted with a RESILIA tissue valve in the COMMENCE trial between January 2013 and March 2016, 458 met the criteria for inclusion in this subanalysis, including 135 in the Pure AR + MAVD group and 323 in the Pure AS group (Figures 1 and E1). The mean duration of follow-up was 5.3 ± 2.2 years. Baseline characteristics differed significantly between the 2 groups (Table 1); notably, compared to patients with Pure AS, patients with Pure AR + MAVD tended to be younger (65.0 years vs 69.0 years; *P* < .0001) and to have a lower risk of predicted operative mortality (Society of Thoracic Surgeons [STS] risk score, 1.1% vs 1.5%; *P* = .0006). The Pure AR + MAVD group had a higher proportion of patients with an LVEF ≤55% (32.5% vs 13.6%; *P* < .0002), larger median LV end-diastolic volume (119.5 mL vs 80.4 mL; *P* < .0001), and larger body surface area (BSA)-corrected LV mass (134.0 g/m^2^ vs 106.2 g/m^2^; *P* < .0001). Baseline covariate balance across the imputed datasets is summarized in Figure E2.

**Table 1:** Baseline patient and echocardiographic characteristics

Variable	All patients (N = 458)	Pure AR + MAVD (N = 135)	Pure AS (N = 323)	*P* value
Age, y, median (IQR)	68.0 (62.0-74.0)	65.0 (55.5-69.5)	69.0 (64.0-75.5)	<.0001
Age group, % (n)				<.0001
<50 y	7.0 (32)	15.6 (21)	3.4 (11)	
50-64 y	25.3 (116)	31.9 (43)	22.6 (73)	
65-80 y	58.7 (269)	48.1 (65)	63.2 (204)	
>80 y	9.0 (41)	4.4 (6)	10.8 (35)	
Female sex, % (n)	29.5 (135)	21.5 (29)	32.8 (106)	.02
BMI, median (IQR)	29.3 (25.9-32.9)	28.2 (25.2-32.4)	29.6 (26.2-33.3)	.01
Bicuspid, % (n)	32.8 (150)	37.0 (50)	31.0 (100)	.21
Endocarditis, % (n)	1.5 (7)	3.7 (5)	0.6 (2)	.03
Mitral stenosis, % (n)	2.8 (13)	3.7 (5)	2.5 (8)	.54
Heart failure, % (n)	22.1 (101)	23.0 (31)	21.7 (70)	.76
EuroSCORE II, %, median (IQR)	1.5 (0.9-2.9)	1.5 (0.7-2.5)	1.6 (1.0-3.1)	.02
STS score, %, median (IQR); total N	1.4 (0.9-2.9); 359	1.1 (0.7-2.1); 101	1.5 (1.0-2.5); 258	.0006
Concomitant group, % (n)				.12
AVR + CABG	17.2 (79)	11.9 (16)	19.5 (63)	
AVR + CABG + other	5.2 (24)	4.4 (6)	5.6 (18)	
AVR + other	16.4 (75)	20.7 (28)	14.6 (47)	
Isolated AVR	61.1 (280)	63.0 (85)	60.4 (195)	
Surgical approach, % (n)				.2
Full sternotomy	80.6 (369)	80.7 (109)	80.5 (260)	
Mini upper sternotomy	17.5 (80)	15.6 (21)	18.3 (59)	
Right thoracotomy	2.0 (9)	3.7 (5)	1.2 (4)	
LVEF, %, median (IQR); total N∗	63.0 (58.0- 68.0); 365	60.0 (53.0- 67.0); 108	64.0 (59.0-68.0); 257	.0003
LVEF group, % (n/total N∗)				.0002
<40%	3.3 (12/365)	5.6 (6/108)	2.3 (6/257)	
40%-55%	15.9 (58/365)	26.9 (29/108)	11.3 (29/257)	
>55%	80.8 (295/365)	67.6 (73/108)	86.4 (222/257)	
Peak gradient, mm Hg, median (IQR); total N∗	62.9 (46.3-77.5); 455	40.6 (17.0-68.3); 133	66.4 (53.7-79.9); 322	<.0001
Mean gradient, mm Hg, median (IQR); total N∗	31.6 (22.7-40.6); 455	21.4 (8.8-34.9); 133	33.6 (27.0-41.6); 322	<.0001
Aortic root diameter, cm, median (IQR); total N∗	3.3 (3.0-3.6); 435	3.6 (3.1-3.8); 128	3.2 (2.9-3.5); 307	<.0001
LV end-diastolic dimension, cm, median (IQR); total N∗	4.6 (4.1-5.2); 366	5.3 (4.6-5.7); 97	4.4 (4.1-4.9); 269	<.0001
LV end-systolic dimension, cm, median (IQR); total N∗	2.8 (2.4-3.4); 366	3.3 (2.7-4.2); 97	2.7 (2.3-3.1); 269	<.0001
LVEDV, mL, median (IQR); total N∗	87.3 (66.6-115.3); 366	119.5 (89.7-173.2); 108	80.4 (61.2- 101.5); 258	<.0001
LVESV, mL, median (IQR); total N∗	29.6 (21.4-45.7); 366	47.0 (27.3-73.3); 108	27.0 (20.0-38.3); 258	<.0001
LV mass, g, median (IQR); total N∗	225.4 (183.7-281.0); 366	266.3 (217.9-332.6); 97	215.7 (180.5- 264.5); 269	<.0001
BSA-corrected LV mass, g, median (IQR); total N∗	111.2 (95.2-138.2); 366	134.0 (104.5- 164.4); 97	106.2 (92.3- 128.0); 269	<.0001

Categorical variables were compared with the χ^2^ test or Fisher exact test if the expected value of any cell was <5. Continuous non-normal variables were compared with Wilcoxon rank-sum test. *AR*, Aortic regurgitation; *MAVD*, mixed aortic valve disease; *AS*, aortic stenosis; *BMI*, body mass index; *IQR*, interquartile range; *STS*, Society of Thoracic Surgeons; *AVR*, aortic valve replacement; *CABG*, coronary artery bypass grafting; *LVEF*, left ventricular ejection fraction; *LV*, left ventricular; *LVEDV*, left ventricular end-diastolic volume; *LVESV*, left ventricular end-systolic volume; *BSA*, body surface area.

∗ Total N represents the total number of patients with available data for the variable.

### Clinical Endpoints

**Figure 2:**
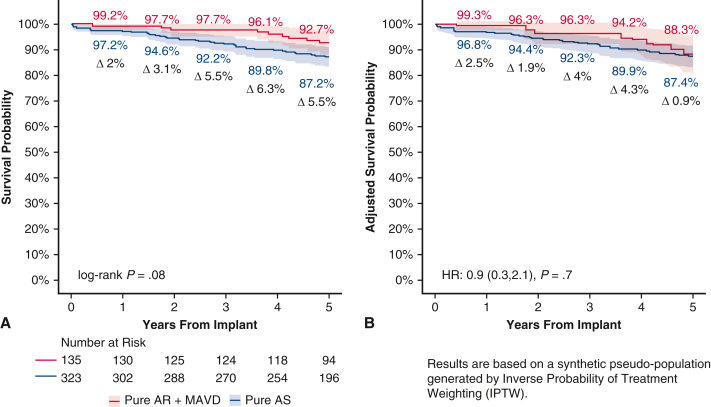
Five-year survival curves for the Pure AR + MAVD group and Pure AS group with 95% confidence interval. A, Unadjusted Kaplan-Meier curves. B, IPTW-weighted Kaplan-Meier curves. *AR*, Aortic regurgitation; *MAVD*, mixed aortic valve disease; *AS*, aortic stenosis.

**Figure E3:**
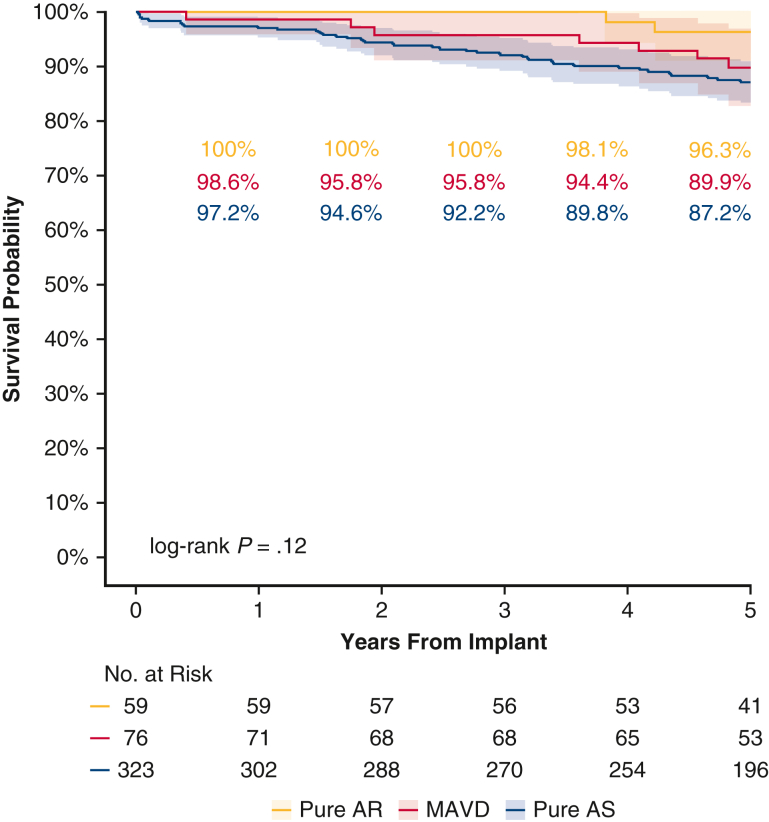
Five-year unadjusted Kaplan-Meier curves of all-cause mortality for 3 patient groups—Pure AR, MAVD, Pure AS—with 95% confidence intervals. *AR*, Aortic regurgitation; *MAVD*, mixed aortic valve disease; *AS*, aortic stenosis.

After adjustment, safety outcomes were similar in the Pure AR + MAVD and Pure AS group (Table 2 and Figure 2). The primary endpoint, pooled IPTW-adjusted Kaplan-Meier estimates at 5 years of freedom from all-cause mortality, also was similar in the Pure AR + MAVD and Pure AS groups (88.3% vs 87.4%; *P* = .67). The IPTW-adjusted hazard of death was 10% lower in the Pure AR + MAVD group; however, this result was not significant (hazard ratio [HR], 0.9; 95% confidence interval, 0.3-2.1; *P* = .70). When evaluating the Pure AR group separately, the 5-year unadjusted survival was 96.3% (Figure E3). No structural valve deterioration was reported in either group during the first 5 postoperative years (Table 2).

**Table 2:** Safety outcomes at 1-year and 5-year (freedom from event)

Outcome	Pooled IPTW-adjusted KM estimates, % (SE)∗				*P* value†
	Pure AR + MAVD (N = 135)		Pure AS (N = 323)		
	1 y	5 y	1 y	5 y	
All-cause mortality	99.3 (0.9)	88.3 (3.7)	96.8 (1.1)	87.4 (2.1)	.67
All reoperations	98.2 (1.5)	97.9 (1.6)	100 (0)	99.0 (0.7)	.43
All bleeding	94.3 (2.6)	91.5 (3.2)	94.8 (1.4)	89.1 (2.1)	.61
Major bleeding	98.3 (1.5)	96.1 (2.3)	96.7 (1.1)	94.4 (1.5)	.53
Endocarditis	97.9 (1.6)	97.6 (1.7)	99.4 (0.5)	97.5 (1.0)	.88
Paravalvular leak	100 (0)	100 (0)	99.0 (0.6)	98.3 (0.8)	.21
SVD	100 (0)	100 (0)	100 (0)	100 (0)	N/A
NSVD	100 (0)	100 (0)	100 (0)	100 (0)	N/A

*IPTW*, Inverse probability of treatment weighting; *KM*, Kaplan-Meier; *AR*, aortic regurgitation; *MAVD*, mixed aortic valve disease; *AS*, aortic stenosis; *SVD*, structural valve deterioration; *N/A*, not applicable; *NSVD*, nonstructural valve deterioration.

∗ SE based on Greenwood's formula. Extreme weights trimmed at the 2.5th and 97.5th percentiles.

† Weighted log-rank test *P* values.

### Echocardiography (Secondary Outcomes)

**Figure 3:**
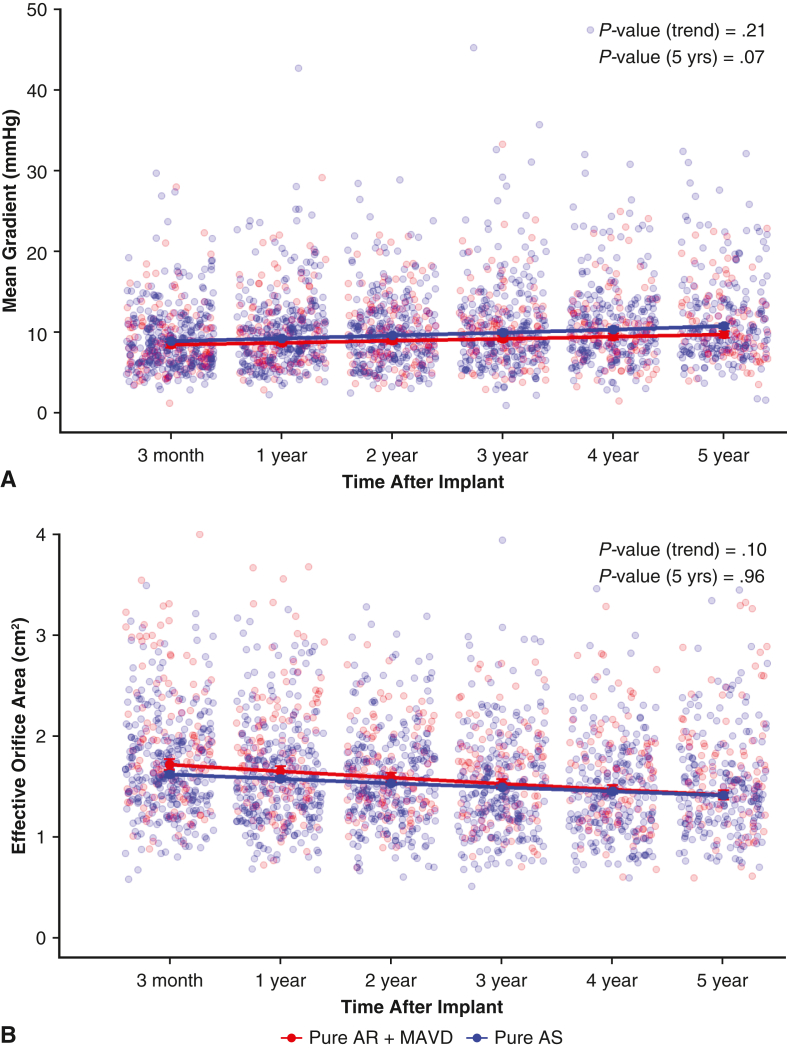
Valve hemodynamics during the 5-year follow-up period. Mean gradient (A) and effective orifice area (B) over time for the Pure AR + MAVD and Pure AS groups. *AR*, Aortic regurgitation; *MAVD*, mixed aortic valve disease; *AS*, aortic stenosis.

**Figure E4:**
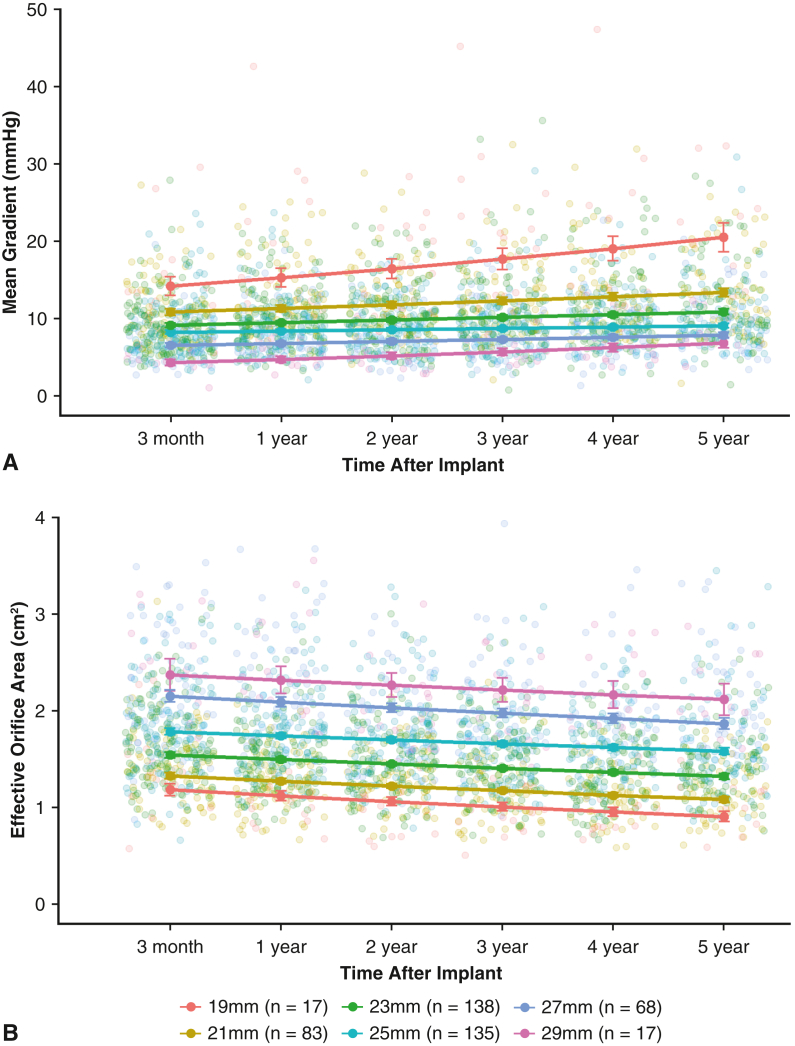
Echocardiography-derived valve hemodynamics over a follow-up of 3 months through 5 years: mean gradient (A) and effective orifice area (B).

Adjusted mean gradients and EOA were clinically stable across both groups through 5 years (Figure 3). For all patients, there was no difference between groups in the rate of change over time for prosthetic mean gradient (*P* = .21) or EOA (*P* = .10). There was minimal increase in the mean gradients for valves of 21 to 29 mm (Figure E4). There was a more appreciable increase in mean gradient in the 19-mm bioprosthetic valves over the 5-year period. Correspondingly, there was a similar small decrease in the EOA during the 5-year period (Figure E4).

**Figure 4:**
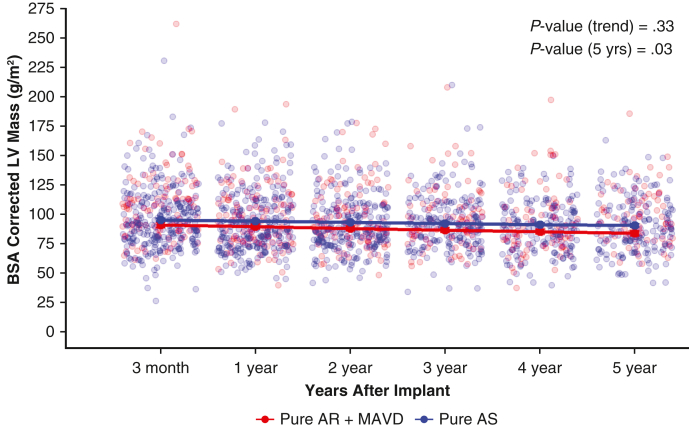
Body surface area–corrected left ventricular mass during the 5-year follow-up period for the Pure AR + MAVD and Pure AS groups. *AR*, Aortic regurgitation; *MAVD*, mixed aortic valve disease; *AS*, aortic stenosis.

**Figure E5:**
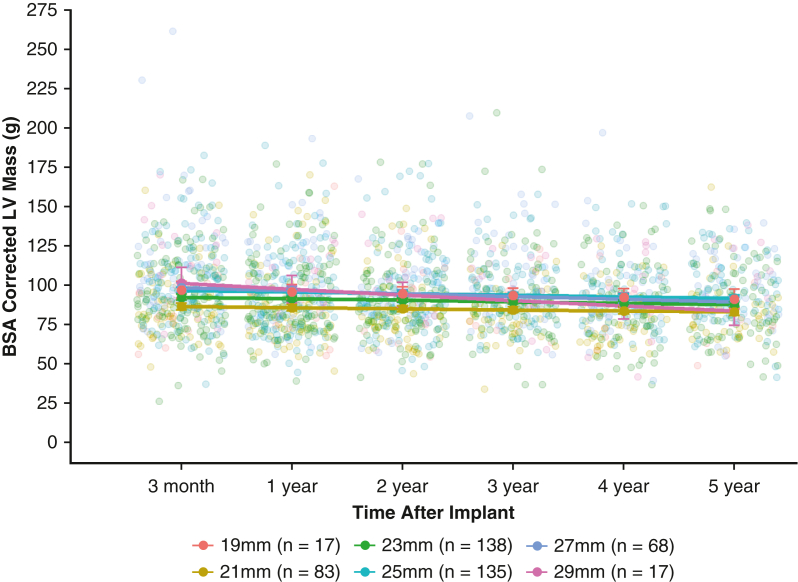
Body surface area–corrected left ventricular mass based on valve size over 5 years.

The adjusted analyses for LV dimensions showed significant LV reverse remodeling in patients with Pure AR + MAVD. BSA-corrected LV mass was greater in patients with AR with or without AS (Figure 4 and Table 3; *P* = .03). The BSA-corrected LV mass for each prosthesis size from 19 to 29 mm showed improvement in LV mass regression (Figure E5). Significant differences were also observed between groups in the postoperative 5-year estimate of LV end-systolic dimension (*P* = .0001), LV end-diastolic dimension (*P* = .003), and LV mass (*P* = .04). Additionally, a greater decrease in LV mass was observed over 5 years in the Pure AR + MAVD group compared to the Pure AS group (Table 3). Of the 420 subjects who had available BMI, EOAi, MG, and DVI data at 3 months, no patient had severe PPM with a mean gradient >20 mm Hg and DVI <0.25.

**Table 3:** Echocardiographic data over 5 years

Variable	Unadjusted (observed data)∗		Adjusted (model includes balancing score)†				*P* value‡
	Baseline		3 mo		5 y		
	Pure AR + MAVD	Pure AS	Pure AR + MAVD	Pure AS	Pure AR + MAVD	Pure AS	
LVEF, %	60.0 (53.0-67.0)	64.0 (59.0-68.0)	64.15 (62.72-65.57)	63.13 (62.20-64.06)	63.23 (61.62-64.85)	61.59 (60.48-62.69)	.11
LVESV, mL	47.0 (27.3-73.3)	27.0 (20.0-38.3)	26.37 (24.20-28.55)	29.31 (27.80-30.82)	27.46 (25.03-29.88)	30.37 (28.65-32.09)	.08
LVEDV, mL	119.5 (89.7-173.2)	80.4 (61.2-101.5)	76.59 (71.55-81.62)	81.26 (78.01-84.51)	77.89 (72.42- 83.36)	82.22 (78.61-85.82)	.22
LVESD, cm	3.3 (2.7-4.2)	2.7 (2.3-3.1)	2.53 (2.41-2.64)	2.68 (2.60-2.75)	2.63 (2.49-2.76)	2.99 (2.89-3.09)	.0001
LVEDD, cm	5.3 (4.6-5.7)	4.4 (4.1-4.9)	4.26 (4.13-4.39)	4.45 (4.37-4.53)	4.36 (4.21-4.51)	4.64 (4.55-4.73)	.003
LV mass, g	266.3 (217.9-332.6)	215.7 (180.5-264.5)	181.38 (170.85-191.92)	191.63 (185.50-197.77)	168.75 (158.32-179.19)	182.32 (175.96-188.67)	.04
BSA-corrected LV mass, g	134.0 (104.5-164.4)	106.2 (92.3-128.0)	90.99 (86.24-95.74)	95.13 (92.38-97.88)	83.78 (79.07-88.50)	90.43 (87.54-93.33)	.03

*AR*, Aortic regurgitation; *MAVD*, mixed aortic valve disease; *AS*, aortic stenosis; *LVEF*, left ventricular ejection fraction; *LVESV*, left ventricular end-systolic volume; *LVEDV*, left ventricular end-diastolic volume; *LVESD*, left ventricular end-systolic dimension; *LVEDD*, end-diastolic dimension; *LV*, left ventricular; *BSA*, body surface area.

∗ Observed data are summarized as median (interquartile range).

† Pooled mixed model results are adjusted for variable baseline value and the logit of the balancing score. Data are estimated marginal mean with 95% confidence interval.

‡ *P* values for differences between groups at 5 years.

### Subgroup Analyses

**Figure E6:**
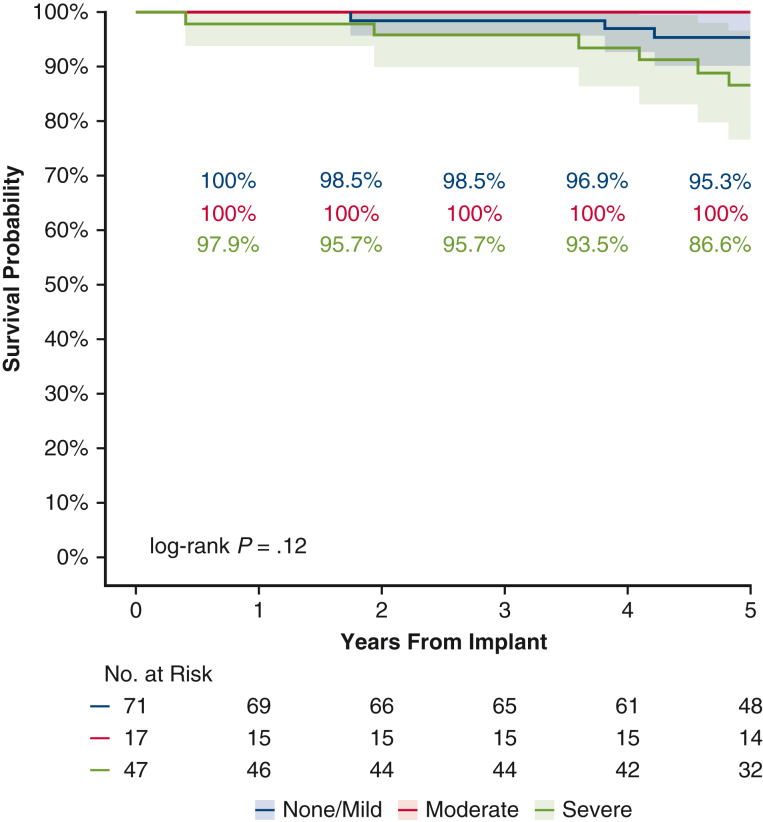
Survival curves in the Pure AR + MAVD group based on the severity of aortic stenosis. *AR*, Aortic regurgitation; *MAVD*, mixed aortic valve disease.

Additional analyses were performed to determine the impact of AS on patients with MAVD. No significant difference in survival was observed among patients with none/mild, moderate, or severe stenosis (*P* = .12) (Figure E6).

All patients in the MAVD group were investigated for pseudo-AS (peak velocity >2.0-2.5 m/s or aortic valve area of >2.0 cm^2^). None of the patients with MAVD had an aortic valve area >2.0 cm^2^. All patients in this group had a peak velocity >2.0 m/s, and no pseudo-AS was noted. Two MAVD patients had a peak velocity between 2.0 and 2.5 m/s; both had missing EOA values at baseline.

For Pure AR + MAVD patients, those with LVEF >55% at baseline showed a decrease (−6.5%; *P* = .003) in LVEF at 5 years compared to the preoperative baseline. This significantly differed for patients with LVEF ≤55%, representing 32% of the group with a median LVED of 5.3 at baseline, in which there was a significant median increase (9.5%) over the study period (51% to 57%; *P* = .0007). Patients with better LVEF at baseline (>55%) continued to demonstrate better LVEF at 5 years compared to patients with poor LVEF (≤55%) at baseline (*P* < .0001).

## Discussion

**Figure 5:**
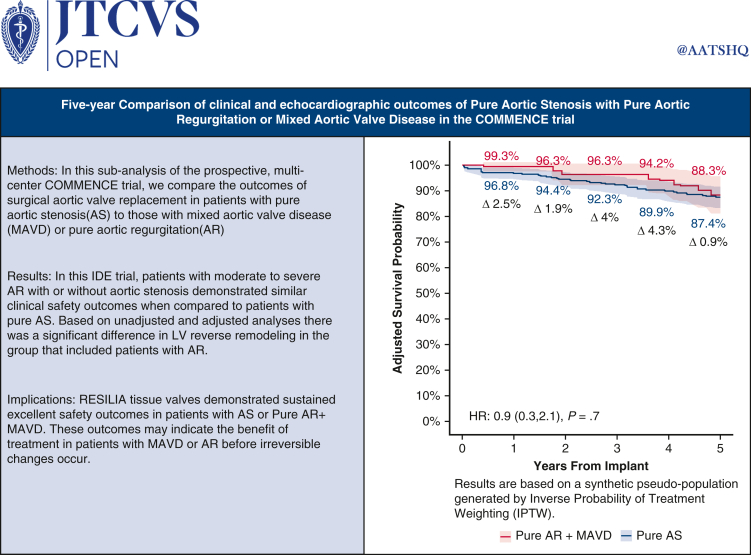
5-year IPTW-weighted Kaplan-Meier curves of all-cause mortality for two patient groups (pure AR + MAVD vs pure AS) with 95%.

This study represents one of the largest assessments of Pure AR or MAVD at 5 years after AVR with echo core lab and CEC-adjudicated data evaluating RESILIA tissue in the modern era. The main findings can be summarized as follows. First, in contrast to prior publications, the 5-year survival was similar in patients with Pure AS and those with Pure AR + MAVD. Second, freedom from all cause-mortality for patients with Pure AR was 96.3% through 5 years, consistent with an STS dataset showing freedom from all-cause mortality of 96.4% at 5 years for patients with an STS predicted risk of mortality <1%.^18^ Third, echocardiographic performance of the valves at 5 years was excellent in both groups, with stable gradients and EOAs. Fourth, there was a sustained reduction in LV mass index in both groups, but more significantly in the Pure AR + MAVD group. Finally, patients with better LVEF at baseline (>55%) continued to demonstrate better LVEF at 5 years compared to patients with poor LVEF (≤55%) at baseline. The current subanalysis of the COMMENCE trial adds to the literature as the first echo core lab and CEC-adjudicated analysis of the Pure AR or MAVD patient population. Figure 5 provides a graphical abstract of the subanalysis.

The existence of MAVD with AR and AS has not been studied comprehensively. Most contemporary clinical trials exclude these patients; thus, the optimal timing for intervention remains unclear. Most recently, Ngiam and colleagues^2^ evaluated 88 of 1188 patients with coexisting AR and AS and noted an increased incidence of adverse outcomes (HR, 1.36) compared to patients with severe AS alone when treated medically. In multivariable Cox regression, coexisting AR remained an independent predictor for poorer outcomes of cardiac failure and mortality at 3 years.^2^ Similarly, Isaza and colleagues^19^ noted a 48.8% mortality in medically-treated MAVD at 5 years. Pathophysiologic mechanisms for poorer outcomes include additional stresses on the left ventricle, which must endure not only volume overload from the AR, but also hypertrophy and LV stiffness from the AS, resulting in reduced compliance and worsening diastolic dysfunction.^4^^,^^20^ Patients with MVAD may have more rapid pathologic remodeling and hemodynamic decompensation and likely need to be treated earlier than isolated AS to prevent irreversible remodeling and long-term consequences of LV overload.^4^^,^^5^^,^^21^

Outcomes associated with AVR in patients with MAVD have not been robust, with most performed as retrospective analyses without adjudicated echo core laboratories or CEC adjudication. There have been mixed results in surgery for MAVD, with some reports noting no increased mortality in patients with preexisting AS with mild or moderate AR (*P* = .19) and others noting significantly worse outcomes in patients with severe AR and severe AS (*P* = .02).^22^ Other studies have shown similar survival in patients with AS compared to patients with AS and moderate to severe AR.^3^ More recent studies have reported improvement in adverse outcomes from AVR for MAVD. Isaza and colleagues found a survival benefit of surgery over watchful waiting in an adjusted Cox analysis (HR, 0.41; *P* < .001), reporting 4.2% survival in the SAVR group at 5.6-year follow-up, compared to 31.9% in the medical arm.^19^ Similarly, we also found excellent survival in our MAVD group, with 88.3% survival at 5 years.

The timing of intervention for pure AR or MAVD remains controversial.^23^ An important tenet remains reversing the deleterious effect of long-term regurgitation on the left ventricle. Currently, TTE with Doppler remains the main modality for detecting regurgitation. However, multiple publications have proposed the significance of cardiac magnetic resonance as a new paradigm for the timing of surgical intervention specifically in patients with AR demonstrating myocardial fibrosis predictive of worse LV recovery.^24^ Hashimoto and colleagues,^25^ in a multicenter retrospective study, demonstrated that cardiac magnetic resonance quantification of LV end-systolic volume indexed and AR severity may identify those at risk of death or incident of heart failure while there were no significant differences observed by echocardiography. Additionally, high levels of brain natriuretic peptide (BNP) or N-terminal pro-BNP predict mortality and cardiovascular events,^24^ and AVR may be warranted in patients with a NT-proBNP ratio ≥3.^26^

Most clinicians still use diminishing LVEF for determining treatment thresholds for AR or MAVD. Our present findings are consistent with prior studies highlighting the benefits of treatment for patients with AR with an LVEF <55%.^27^, ^28^, ^29^, ^30^ The present study also demonstrates that patients treated with preserved LVEF (>55%) at baseline continued to demonstrate better LVEF at 5 years compared to those with a baseline LVEF ≤55%. A low LVEF in patients with significant AR is an important predictor of death following surgery, as well as of long-term survival and functional status. There should be continued vigilance in treating symptomatic patients with severe AR prior to deterioration of the LVEF, which may lead to myocardial preservation.

### Limitations

Although we report excellent outcomes for AR and MAVD patients in the COMMENCE trial, this is a post hoc analysis and was not designed for or powered for this comparison. Moreover, the study cohort contained a relatively low number (n = 59) of patients with pure AR. Owing to the observational nature of the study design, we attempted to correct for potential selection bias in the analyses by using a balancing score and IPTW-adjusted survival analyses; nonetheless, additional confounders may exist that were not measured in the study. Additionally, high-volume surgical centers enrolled a highly selected group of patients in this study, and thus the outcomes might not be generalizable. The patients were a heterogeneous group with mild to severe AS. Although we accounted for pseudo-AS and found that no patients met the criteria, we did not investigate whether MAVD patients had symptomatic AS or AR.

## Conclusions

This dataset represents a prospective, echo core lab, and CEC-adjudicated data highlighting outcomes following surgical AVR for patients with MAVD or Pure AR. The RESILIA aortic bioprosthesis has continued to demonstrate excellent safety outcomes in patients with pure AS, MAVR, and pure AR. Timely consideration of AVR may aid LV reverse remodeling for patients with AR. Continued long-term follow-up in these patient populations is needed for this innovative AV bioprosthesis.

### Webcast

You can watch a Webcast of this AATS meeting presentation by going to: https://www.aats.org/resources/five-year-comparison-of-clinic-7215.

**Figure:**
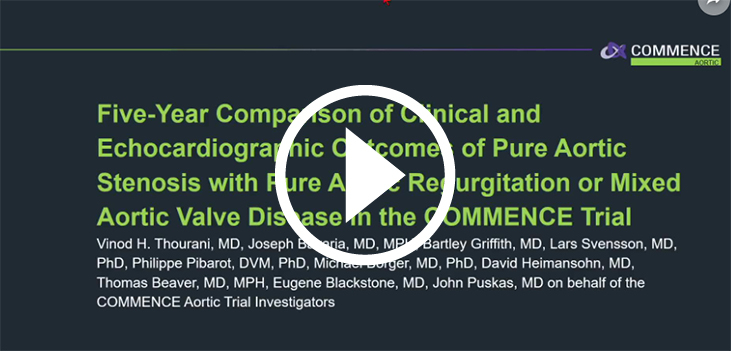

## Conflict of Interest Statement

V.H.T. reports equity in Dasi Simulations and consulting or research for Abbott Vascular, Artivion, Atricure, Boston Scientific, CroiValve, Edwards Lifesciences, Highlife, Innovalve, Jenavalve, Medtronic, and Trisol. All other authors reported no conflicts of interest.

The *Journal* policy requires editors and reviewers to disclose conflicts of interest and to decline handling or reviewing manuscripts for which they may have a conflict of interest. The editors and reviewers of this article have no conflicts of interest.
